# Factors Contributing to Early Recovery of Urinary Continence Following Radical Prostatectomy: A Narrative Review

**DOI:** 10.3390/jcm13226780

**Published:** 2024-11-11

**Authors:** Bara Barakat, Boris Hadaschik, Mulham Al-Nader, Samer Schakaki

**Affiliations:** 1 Urology Centre, Albertusstraße 17, 41061 Moenchengladbach, Germany; 2 Department of Urology and Pediatric Urology, Hospital Viersen, 41747 Viersen, Germany; 3 Department of Urology, University Hospital Essen, 45147 Essen, Germany; boris.hadaschik@uk-essen.de (B.H.); mulham.al-nader@uk-essen.de (M.A.-N.); 4 Department of Urology, Hospital Kassel, 34125 Kassel, Germany; samerschakaki@hotmail.de

**Keywords:** stress urinary incontinence, radical prostatectomy, membranous urethral length, nerve-sparing, retzius-sparing radical prostatectomy

## Abstract

Stress urinary incontinence (SUI) is a common condition in patients following radical prostatectomy (RP), which has a significant impact on all aspects of quality of life and is associated with significant social stigma. The factors that improve urinary incontinence in patients following surgery remain controversial. The aim of our narrative review was to identify and synthesise the latest evidence on pre-, intra- and post-operative factors and predictors that contribute to early continence recovery after RP. In this narrative review, primary resources were identified by searching PubMed, EMBASE and Medline, and secondary resources were collected by cross-referencing citations in the relevant articles. We started our review by searching for systematic reviews of factors and predictors that contribute to early recovery of urinary continence after RP. We then reviewed societal guidelines such as the American Urological Association and European Urological Association guidelines on male urinary incontinence. This review focuses on the pre-, intra and postoperative factors that influence postoperative SUI after RP, as well as highlighting modifications in surgical techniques that lead to early continence recovery. Increasing age, higher BMI, shorter membranous urethral length (MUL), and larger PV are independent prognostic factors for SUI within 3 months after RP. Factors such as modified surgical technique preservation of anatomical structure lead to influence postoperative early continence recovery. SUI after RP is influenced by various factors. These factors include not only anatomical landmarks and patient-related factors such as age, BMI, length of MUL and prostate volume, but also prior transurethral resection or laser enucleation of the prostate, the surgeon’s expertise, the surgical approach and NS technique.

## 1. Introduction

Prostate cancer is a serious health concern for men worldwide. It is the second most frequent cancer diagnosis made in men and the fifth leading cause of death globally [1,2]. Radical prostatectomy (RP) is a standard treatment option with curative intent performed for localized prostate cancer. During the last decades, great efforts have been made to develop technical modifications of the classical open surgical technique to improve oncological and functional outcomes after surgery. Advances in surgical techniques have reduced the rate of post-prostatectomy incontinence (PPI). However, the burden of PPI remains high and is expected to increase due to the increasing number of procedures performed [3]. The incidence of PPI of various types and degrees is higher than previously thought and can affect up to 96% of patients [4]. Therefore, the most common cause of persistent SUI in men is RP, with several studies reporting a progressive return of continence up to one year after RP, with SUI rates ranging from 6.8 to 47% at 12 months [5,6]. In a recently published study by Kowalski et al., the rate of incontinence after RP was 57% at 12 months [7]. Other causes of SUI in men include severe pelvic trauma and neuropathy affecting the external sphincter mechanism. Post-operative dysfunction, such as SUI, is a major concern for patients undergoing RP for prostate cancer. Studies have shown that SUI can have a significant negative impact on the social life of affected patients, including reduced quality of life with abstinence from daily activities and increased feelings of isolation or embarrassment [8,9]. In addition, SUI has financial consequences for these individuals and for healthcare systems [10].

The exact aetiology of PPI has not been completely understood. However, a deeper understanding of the anatomical structure, prostate anatomy and function of the pelvic floor involved in urinary continence is essential for comprehending the factors that contribute to its impairment postprostatectomy. The urethral sphincter complex, which includes the external urethral sphincter and the pubourethral ligaments, plays an important role in maintaining urinary control. In recent years, changes in surgical techniques and technological advances have improved the functional and oncological outcomes of RP [11]. The preservation of anatomical structures (endopelvic fascia, arcus tendineus, puboprostatic ligaments, Santorini plexus and the neurovascular bundle, Denonvilliers’ fascia and pelvic floor levator ani muscles) led to better continence rates [12]. Several factors and limitations must be considered when interpreting the assumed outcomes of RP, including pre-, intra-, and postoperative factors. The following manuscript aims of our narrative review were pre-, intra and postoperative factors and predictors of early continence recovery after RP.

## 2. Materials and Methods

In this narrative review, primary resources were identified by searching PubMed, EMBASE and Medline, and secondary resources were collected by cross-referencing citations in the relevant articles. Our methodology is summarised in Table 1. We began our review by searching for systematic reviews of factors contributing to early urinary continence after RP. We then reviewed societal guidelines such as the American Urological Association and European Urological Association guidelines on male urinary incontinence. This gave us a broad selection of the scientific literature to date, which we have summarised in this review. As this is a narrative review summarising the results of different studies, the definitions and outcomes of interest may vary between articles. This is a common problem in the SUI literature, as studies lack standardisation in both the classification of SUI and the measurement of outcomes. In addition, this review aims to provide the urologist with an overview of the factors that influence urinary incontinence following such procedures. The decision to undergo RP should be a joint one between the patient and the urologist.

### 2.1. Pathophysiology of Urinary Incontinence

An understanding of the anatomical structures involved in micturition and urinary continence is important. The mechanism of urinary continence depends on the normal functioning of several key anatomical structures, including the bladder, urethra, and urethral sphincter, and the nerves that innervate these components. Knowledge of the exact location and function of these structures is essential for surgical procedures aimed at maintaining or restoring continence.

The pathophysiology of post-radical prostatectomy stress urinary incontinence (SUI) has a multifactorial aetiology involving both anatomical and functional factors. Based on the current literature, the most important factors contributing to post-RP incontinence are changes in anatomy and surgical technique [11,13]. Surgical removal of the prostate can damage the anatomical structures or nerves involved in urinary continence, resulting in varying degrees of impairment, including postoperative SUI. There are three main nerves that are important in the mechanism of continence: the pudendal nerve, the autonomic supply of the internal sphincter via the hypogastric nerves and pelvic plexi, and the neurovascular bundle, which has been shown to provide some innervation of the membranous urethra [14]. Several studies demonstrated that the maximal protection of anatomical structures during RP, such as the endopelvic fascia, tendinous sheath, puboprostatic ligaments and neurovascular bundle, have been identified as important factors for continence after RP [13,14]. Dysfunction of the nerves innervating the pelvic floor or urethral sphincter may cause stress incontinence after RP [13,14]. Common anatomical changes include the inferior displacement of the bladder junction and the proximal membranous urinary tract stump in the pelvis. Prostatectomy removes the smooth muscle of the prostate and urethra, and postoperative anastomotic stricture results in increased urinary tract stiffness decreased urinary tract elasticity and ultimately reduced urinary pressure during pelvic floor muscle contraction [14]. In addition, sphincter incompetence is a result of damage to the sphincter itself and the supporting structures, the nerves, which can recover over time [15]. This study suggests that preservation of fascial support lateral to the urethra and prostate protects neurovascular structures important in improving post-prostatectomy urinary continence [16].

### 2.2. Patient-Related Preoperative Prognostic Factors for SUI Following RP

Several risk factors have been suggested to increase the likelihood of SUI following RP, including preoperative comorbidities (age, body mass index, preoperative urinary incontinence, length of membranous urethra and history of previous prostate surgery) and intraoperative factors (surgical technique and surgical experience).

Age: Age may have a negative impact on continence recovery after surgery, as older patients have other potential risk factors such as larger prostate size, a higher incidence of overactive bladder, or comorbidities such as diabetes mellitus when compared to younger patients. It plays an important role in continence recovery and in pre-existing urinary continence. Several studies highlighted the impact of age on continence outcomes after prostatectomy. In our recently published study, we analysed data from 154 patients who underwent RP. Our data suggest that age has a significant impact on early continence recovery [17]. Similarly, Lavigueur-Blouin et al. demonstrated the early continence recovery after RARP [18]. The authors concluded that age is an independent predictor of early continence after RP. Men of advanced age and those with significant lower urinary tract symptoms should be advised of the increased risk of UI prior to RARP [18]. The outcomes of RARP in older men were compared to those in younger men in a study by Greco et al. [19]. The researcher revealed that continence rates at 1, 3 and 12 months were similar between the two groups; however, the rate of incontinence in the older group at 6 months post-surgery was significantly higher. A possible cause for this is poor endothelial function, which impairs the vascular supply of the neurovascular bundles [19]. 

In a study based on more than 8000 patients, Mandel P et al. found that the 1-year continence rate decreased with patient age, from 93.2% in patients <65 years to 86.5% in patients ≥75 years [20]. Age-related factors, such as decreased muscle tone and elasticity of the urinary sphincter, may contribute to a higher risk of SUI in older individuals.

BMI: In fact, there is a lot of discussion in the literature on the impact of BMI (Body Mass Index) on postoperative continence following RP. It is believed that postoperative SUI is associated with higher BMI. However, for the hypothesis there is no clear explanation [21]. Due to obesity, adipocytokines are secreted, which may lead to an increase in sympathetic tone, which has a proliferative influence on prostate cells and a negative impact on the lower urinary tract [22]. Yong Wei et al. indicated in their meta-analysis that BMI is a significant factor influencing early continence recovery [23]. However, there was no significant association between obesity and SUI at 24 months in patients following RP. However, other studies have shown conflicting results, with no significant correlation between BMI and postoperative SUI. Mandel et al. showed in 2471 RP patients that BMI was an independent risk factor for functional outcomes after RP and that high BMI values were predictors of worse SUI outcomes at the 12-month follow-up [24]. These studies suggest that predictors such as age, preoperative continence condition and surgical technique may have a greater impact on SUI outcomes than BMI alone. Controversially, Xu et al. demonstrated similar SUI outcomes between obese (BMI > 30 kg/m^2^) and non-obese (BMI < 30 kg/m^2^) RP patients, claiming that RP appears to provide satisfactory functional outcomes even in obese men [25]. Further research is needed to better understand the relationship between BMI and postoperative SUI after RP. In the clinical setting, healthcare professionals should consider multiple factors, including BMI, when assessing the risk of postoperative SUI and provide individualised counselling and support to patients based on their unique circumstances.

Membranous urethral length (MUL): Preservation of the MUL by accurate dissection of the prostatic apex during surgery is recommended for early continence recovery following RP [26]. The MUL, as measured by magnetic resonance imaging (MRI) with almost 400 patients, has consistently been a strong predictor of urinary continence recovery following RP [27,28,29]. Our recently published study demonstrated that the likelihood of continence recovery increases with membranous urethral length and decreases with age, BMI and lack of nerve-sparing [17]. In our analysis, preoperative MUL >15 mm (95% CI 1.28–1.33; *p* = 0.03) and postoperative MUL >14 mm (95% CI 1.2–1.16; *p* = 0.05) were significantly associated with early continence recovery at 3 months postoperatively [17]. According to a systematic review by Mungovan et al., longer preoperative MUL was associated with early continence recovery after RP [30].

History of previous prostate surgery and high prostate size: Postoperative UI following RP in patients with a history of previous surgery has been investigated. Several studies investigated this relationship and provided insights into the potential impact of previous surgery on continence outcomes after RP. A recently published study compared a total of 368 patients undergoing robot-assisted radical prostatectomy (RARP) with prior transurethral resection or laser enucleation of the prostate to 4945 patients undergoing RARP without transurethral resection or laser enucleation of the prostate [31]. The authors concluded that transurethral resection or laser enucleation of the prostate has a negative effect on erectile function and recovery of urinary continence [31]. Patients undergoing RP with a history of previous prostate surgery are more likely to develop postoperative UI due to pre-existing LUTS [31]. It is important to recognise that the impact of pre-existing LUTS on post-operative continence outcomes depends on several factors, including the severity of LUTS, the surgical technique used for deobstruction and the volume of the prostate. According to the results of the study by Mandel et al., higher prostate volume was associated with adverse effects on postoperative UI after prostate surgery, both in the short term (1 week–3 months) and in the long term (6–12 months) [20]. Specifically, the shape and volume of the prostate may have a significant impact on both the length of the urethra and the dissection of the bladder neck for several reasons, including the potential presence of a central lobe, a greater distance between the bladder and urethra and a significant risk of denervation due to reduced mobility of the large gland.

### 2.3. Preoperative Pelvic Floor Muscle Training (PFMT) to Optimise Continence Following RP

The postoperative standard of care with postoperative physiotherapist-guided PFMT is recognised as a multidisciplinary modality for continence recovery after RP. Improvements in postoperative PFMT have contributed to significant reductions in incontinence and improvements in quality of life for affected patients [32,33]. Traditional interventions to improve UI after RP have typically focused on postoperative PFMT provided during the postoperative period (rehabilitation). Prehabilitation is a relatively new concept that is increasingly important in improving functional outcomes. The aim of preoperative interventions is to improve postoperative functional capacity and recover urinary continence, patient well-being and quality of life [34,35].

However, it is still difficult to determine the effectiveness of pPFMT due to a lack of randomised controlled trials (RCTs). The duration of a patient-centred prehabilitation programme is partly determined by each patient’s waiting time for surgery, defined as the time from diagnosis of prostate cancer to the date of surgery.

### 2.4. Intraoperative Factors and Modifications of Surgical Techniques

Surgical techniques: During the last decades, great efforts have been made to develop technical advances and modifications of the classic open RP technique, including minimally invasive techniques, with the aim of improving oncological and functional outcomes and minimising patient morbidity after surgery. Since its development and introduction, RARP has been suggested to have a major advantage over open RP in that it better preserves the anatomical structure. There have been a number of modifications and refinements in surgical techniques to optimise continence after RARP. These include modifications in apical dissection to maximise urethral length and maximise preservation of periurethral structures [36], preservation of the anterior puboprostatic complex (anterior reconstruction) [37] and combinations of these strategies.

An analysis of hospital data shows that RARP reduces hospital stay and blood loss, but there are no clear oncological, functional or quality-of-life benefits [33]. According to a prospective study by Geraerts et al., patients who underwent RARP tended to recover urinary continence earlier than those who underwent ORP [38]. However, there was no statistically significant difference in continence rates in the long term (12 months). Similarly, O’Neil et al. showed in a population-based study that there was no statistically significant difference in continence rates in either group (RARP vs. ORP) at 12 months [39]. It is important to remember that different aspects, such as the surgeon’s experience, the patient’s characteristics and tumour characteristics, may influence the choice of surgical approach and particular technical adjustments. 

The published study by Haese et al., comparing the two surgical approaches RARP vs. ORP in terms of oncological and functional outcomes, found that both surgical procedures, performed in a large centre by the same surgeons, achieved excellent comparable oncological and functional outcomes [40]. The recently published work by Di Bello et al. reported that the presence of prostate lobe asymmetry negatively affected the recovery of 3 and 12 months of continence in prostate glands ≤40 mL [41].

**Figure 1:**
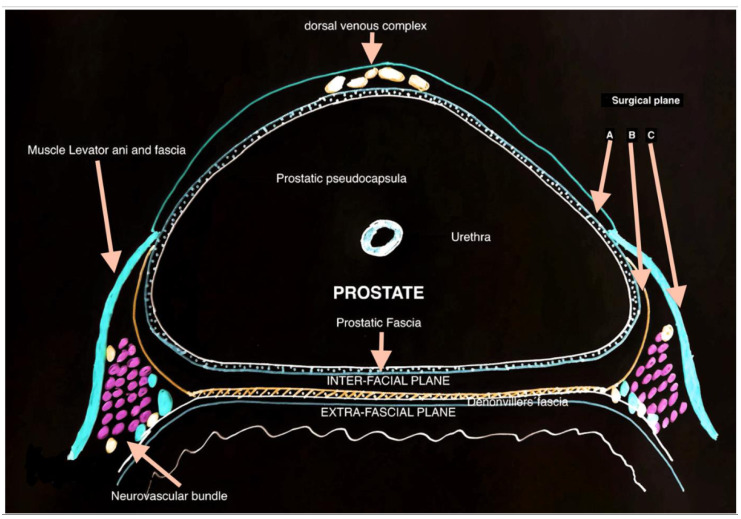
The surgical anatomy of the prostate and axial view of prostatic fascial anatomy. A—intrafascial plane, B—interfascial plane, and C—extrafascial plane with no preservation of neurovascular bundle.

Nerve-sparing approach: Nerve-sparing (NS) approaches preserve the neurovascular bundles responsible for erectile and part of urinary control. This technique reduces the risk of nerve damage, thereby enhancing postoperative continence. There is robust data for nerve-sparing compared to more radical surgical techniques, suggesting that preservation of the vascular and nerve bundles improves postoperative functional outcomes [35]. A meta-analysis of 13,749 patients showed that NS approaches had significantly higher rates of urinary continence recovery at 6 months post-operatively than those who underwent non-nerve-sparing approaches [42]. Two methods are described for NS: the intrafascial and interfascial approaches. In intrafascial dissection, the working plane remains within the prostatic fascia on the anterolateral and posterolateral aspects of the prostate and anterior to the Denonvilliers fascia [43] (Figure 1).

**Figure 2:**
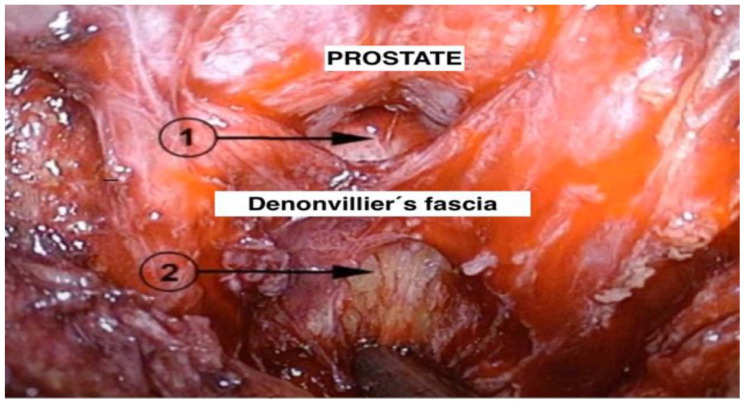
Intraoperative overview, posterior dissection; 1 = intrafascial plane and 2 = extrafascial plane with no preservation of neurovascular bundle.

On the other hand, intrafascial dissection may leave the neurovascular bundle completely intact (Figure 2). There is a risk of iatrogenic capsular injury with this technique, which is associated with a positive surgical margin. Several studies investigated the effect of different fascial planes of the NS on the functional outcome of RP. In a prospective study of 430 patients, Khoder et al. compared intrafascial and interfascial RP. The authors conclude that intrafascial prostatectomy offers better functional outcomes compared to the interfascial approach without compromising oncological outcomes one year after surgery [44]. Another pooled meta-analysis of functional outcomes showed that intrafascial NS prostatectomy could provide patients with earlier return to continence and better erectile function at 1, 3 and 6 months compared to the conventional interfascial approach [45]. Stolzenburg et al. compared the outcomes of interfascial and intrafascial NS techniques and concluded that the intrafascial NS technique was associated with significantly better continence outcomes at 3 and 6 months after surgery compared to the interfascial approach [46].

Unilateral versus bilateral NS: Unilateral versus bilateral nerve-sparing has been investigated in various studies. The benefit of functional outcomes between unilateral and bilateral nerve-sparing approaches appears to be a topic of debate and controversy. While Greco et al. reported that patients undergoing laparoscopic intrafascial NS-RP with bilateral NS had significantly higher efficacy rates than the unilateral group [19]. Finley et al. concluded that there was no significant difference between unilateral and bilateral NS in RARP [47]. Regarding the effect of unilateral and bilateral NS-RP on functional outcomes, further studies may be needed to clarify the controversy surrounding this topic.

Development of anatomical classification of NS: Several studies focused on developing anatomical grading systems for NS during PR to assess the extent and quality of NS during surgery. These grading systems aim to provide a more objective assessment to evaluate the level of nerve preservation and guide surgeons in optimising functional outcomes while balancing oncological outcomes. Tewari et al. proposed a novel four-stage stratification to preserve neural vascular bundles using veins located on the lateral aspect of the prostate as landmarks [48]. Complete intrafascial dissection between the periprostatic veins and the pseudocapsule of the prostate is classified as grade 1 in the Tewari classification system. Grade 2 dissection is performed directly over the veins and is equivalent to interfascial dissection. Grade 3 dissection leaves more tissue over the veins and prostate. Finally, grade 4 corresponds to extrafascial dissection [49]. To define the dissection planes, Schatloff et al. in 2016 presented a five-degree stratification using the landmark artery running on the lateral side of the prostate as a reference point. According to the Schatloff grading system, the neurovascular bundles (NVBs) are not spared in grade 1 dissection, which involves extrafascial dissection. In a grade 2 dissection, the NVB is dissected a few millimetres to the side of the artery. In a grade 3 dissection, the plane of the NS is created laterally to the landmark artery, and the artery is clipped at the level of the prostate pedicle. In a grade 4 dissection, the prostate pseudocapsule is dissected sharply between the landmark artery and the prostate pseudocapsule over the NVBs. Finally, a grade 5 dissection is completed medial to the landmark artery just outside the prostate fascia and corresponds to maximal NS (full intrafascial dissection), in which the prostate and NVBs can be separated without the necessity for sharp dissection [49].

Retzius-sparing approach: In 2010, Galfano et al. described a surgical technique to preserve all the anatomical structures (pelvis, arch tendons, pubic-rectal ligaments, Santorini plexus and vascular bundles) to improve functional outcomes following RP; this technique is called “retzius—sparing radical prostatectomy (RS-RP)” [50]. During the RSRP, the prostate would be removed posteriorly in a completely intrafascial plane without damaging any anatomical structures (santorini plexus, puboprostatic ligaments, arcus tendineus and levator ani muscle). Our recently published meta-analysis, which included RCTs and six prospective studies, suggests that RS-RP is more effective in maintaining urinary continence than RARP and ORP. Overall and major postoperative complication rates appear to be similar [51]. On the downside, PSM rates were statistically significantly higher for localised ≤pT2 tumours after RS-RARP [51].

Maximum preservation of membranous urethral length: Preservation of the intraprostatic portion of the membranous urethra, as well as the external urethral sphincter, is critical to achieving maximum urethral length. Early return to continence is increased by fully functional MUL preservation with modified apical preparation maximal preservation of the intraprostatic membranous urethral segment. The most important step in preserving maximum MUL without increasing the risk of positive apical surgical margins is to accurately identify the junction between the MUL and the prostatic apex [18]. Schlomm et al. described that full functional MUL was preserved by performing an apical dissection strictly along anatomical landmarks, respecting the individual length of the intraprostatic portion of the urethral sphincter. Anatomic fixation of the sphincter was achieved by thorough preservation of the pelvic floor and anatomic restoration of the Mueller’s ligaments [52]. Schlomm et al. analysed 406 RP patients with maximal urethral length preservation and compared the results with 285 patients who underwent RP without preservation of MUL. Continence rates at 1 week after catheter removal were 50.1% in the first group and 30.9% in the second group, but no statistically significant difference was found at 12 months [52].

Anterior and posterior reconstruction: The effect of anterior and/or posterior reconstruction in RP on functional outcomes, particularly urinary continence, has been investigated in several studies. Sessa et al. evaluated the implementation of surgical techniques advocating anterior and/or posterior fascial reconstruction on functional outcomes after RP. The authors suggested that the implementation of surgical techniques could improve the early continence rate after RARP. However, the authors point out that in experienced hands, most patients fully recover urinary continence at mid-term follow-up (17 months) after RARP is performed without specific anterior or posterior reconstruction techniques [53]. According to Patel et al., anterior reconstruction may improve functional outcomes in patients undergoing RARP at 3 months, but continence rates at 6 and 12 months were identical to those in the control group [54]. Rocco et al. presented a procedure based on posterior rhabdosphincter reconstruction. This technique allows the neutral position of the rhabdosphincter [55]. In addition, the published meta-analysis by Wu et al. highlighted the potential benefits of total anatomical reconstruction (anterior and posterior) in improving early urinary continence outcomes [42]. Finally, a large prospective study by Tan et al. demonstrated a statistically significant advantage for the total reconstruction approach compared to anterior and conventional approaches [56]. These findings were supported by a randomised controlled trial that showed a significant benefit in functional outcomes at the 1-month follow-up compared to a standard approach [56].

## 3. Conclusions

To summarize, PPI may be influenced by various factors. These factors include not only anatomical landmarks and patient-related factors such as age, BMI, length of MUL and prostate volume but also prior transurethral resection or laser enucleation of the prostate, the surgeon’s expertise, the surgical approach and NS technique. The development of numerous intraoperative approaches to improve outcomes after RP has been made possible by improvements in surgical technique over the past decade. Understanding these factors is essential to increase continence rates and quality of life for patients. Factors such as surgical technique, pelvic floor preservation, NS and preoperative parameters have been studied as potential contributors to post-prostatectomy incontinence, but further research is needed to determine the exact causes, interactions and mechanisms of different nerve-sparing techniques. There is still much to be known regarding the male continence mechanism, the role of specific structures in maintaining continence and the precise aetiology of PPI. One of the main limitations of the included studies is the definition of SUI and the outcomes of interest, which can vary from article to article. This is a common problem in the SUI literature, as studies lack standardisation in both the classification of SUI and the measurement of outcomes. Individual counselling and careful patient selection for radical prostatectomy, which is the only option to cure localised prostate cancer, are key to avoiding decision regret.

## Figures and Tables

**Table 1:** Search methodology.

Items	Specification
Data of search	2 February 2024–15 August 2024
Databases and other sources searched	PubMed, EMBASE, Medline—search
Search terms used	stress urinary incontinence, radical prostatectomy, age, BMI, Membranous urethral length, Nerve-sparing, retzius-sparing radical prostatectomy
Timeframe	To present
Inclusion and exclusion criteria	Full, English-language manuscripts, when available/preferred
Additional considerations, if applicable	Our search primarily identified previous reviews on the topic and then expanded based on findings within primary sources and societal guidelines

## Abbreviations

Stress urinary incontinence—SUI, radical prostatectomy—RP, post-prostatectomy incontinence—PPI, open surgical technique—ORP, prostate volume—PV, body mass index—BMI, urinary tract symptoms—LUTS, membranous urethral length—MUL, lower urinary tract symptoms—LUTS, urinary incontinence—UI, robot-assisted radical prostatectomy—RARP, nerve-sparing—NS, and retzius-sparing radical prostatectomy—RS-RP.
